# Letter from Mr. Lawson Tait

**Published:** 1894-02

**Authors:** Lawson Tait

**Affiliations:** The Crescent, Birmingham, Eng.


### Correspondence.

*Letter from Mr. Lawson Tait.—He comments in detail on Dr. F. Byron Robinson's paper in the December* JOURNAL, *entitled, "What Kills After Laparatomy — Anesthetic, Nephritis or Infection?"—He begins by protesting against the use of the word laparatomy.—How he discovered that ether arrests the flow of urine.—Interesting history relating thereto.—English practitioners and coroners' inquests.—How chloroform and ether kill.—"Ether mixture far better and safer than chloroform."—Reply to Dr. Robinson's question concerning infection deferred until another communication.*

SIR—In the number of your valuable JOURNAL for the current month, I have just read an interesting paper by my friend and former pupil, Dr. F. Byron Robinson, which requires a little notice, for I think he is hardly fair to me, or to my brethren on this side of the Atlantic, when he says that few of us engaged in abdominal surgery give much attention to the kidney in their work.

His paper has a most suggestive interrogatory title, "What Kills Patients After Laparatomy—Anesthetic, Nephritis or Infection?" Dr. Robinson hardly answers his own question, nor does he make quite clear his intention in asking it, but it is sufficient for my present purpose that he does ask it, and that he does so without that full information which I thought he possessed after his long residence with me. I, therefore, desire to supplement some of his remarks.

I need not stop again to protest against the continued use of the word "laparatomy," which can be used properly for operations involving incisions made in the flank, and cannot be extended to anything like an ordinary ovariotomy, and I therefore pass at once to the question at issue, rejecting this objectionable phrase.

In order to place myself historically right, let me say that it is now about twelve years since I made the discovery that the anesthetic administration of ether in the human subject may—certainly does, in some instances,—completely arrest the flow of urine during its administration. The discovery was made by accident in a case of great rarity, a uretero-uterine urinary fistula, where I failed time after time because I missed the fistula. This was due to the fact that I used ether and therefore thought I had closed the aperture, when I had only temporarily arrested the flow of urine at the very time when I most needed the guidance of its misdirected current. I then used chloroform, closed the fistula properly and cured the patient.

In operating on several bladder cases immediately after this incident, I had opportunities of confirming the experience, and these facts set me thinking. At that time a great enthusiasm had set in for the use of ether, a prejudice had steadily grown against chloroform, and I had, unfortunately, been carried away with the stream. But my new fact set me thinking, and my thoughts drove me into a careful research of a number of fatal cases of operation where no complications of the operation could account for the deaths, but where the fatal results were found to be due to pulmonary and renal lesions, some known to have been in existence before the operation, others not suspected or not possible as antecedents.

I appealed to my friend and colleague, Dr. Robert Saundby, now known as one of our greatest authorities on renal pathology and then our acting pathologist, for assistance, and that was promptly and abundantly given. He had known for years that degenerative changes in the kidney were very commonly associated with all abdominal tumors. Dr. Saundby and I had discussed the fact often before 1878. I knew perfectly well that many operators refused to undertake cases where the coarse test of the presence of albumin in the urine seemed to indicate serious kidney disease. But I had steadily set my face against ever refusing to operate on any case whatever, and, therefore, disregarded this apparently ominous sign. The result was that I scored a great number of brilliant successes in cases refused by men then at the head of my department. But I met with equally disastrous failures in cases where I could not see that anything was wrong with the operation and where Dr. Saundby and, subsequently, Dr. Foxwell steadily reported "kidney degeneration and pulmonary edema."

Then two cases occurred which put a great light on the whole thing, a light which ought to have been admitted before, and would have been recognized long before, but for that most unfortunate habit we adopt of moving restlessly about in streams of fashion, giving to no proceeding and to no plan a systematic and logical investigation. The cases were, briefly, these: A young Irish girl came to me with an enormous ovarian tumor, which had been tapped over and over again, the radical cure having been refused to her by no less than five leading surgeons in Great Britain alone. Her legs were so enormously swollen that no kind of joint flexion was possible, and no vaginal examination could be made, on account of vulvar edema. She passed only about twenty ounces of urine in each twenty-four hours, and quite half of that was albumin. According to our notions at that time, nothing could be more unpromising, but I undertook the operation. On thinking over the special conditions of the case, I remembered Simpson's great belief in chloroform as a remedy for the extreme conditions associated with albuminuria, especially the eclampsia of the puerperal woman and the convulsions of post scarlatinal nephritis. I determined to give this case chloroform, and she got well without an interruption, the albuminuria disappearing as rapidly as her convalescence. In three months she was a strong and perfectly healthy woman.

The other case was that of a strong young woman of thirty, with a rapidly growing soft edematous myoma. All her visceral functions were healthy, so far as could be determined, before the operation. I did hysterectomy, the patient being under the influence of ether. She passed very little and highly albuminous urine after the operation, and she never drew an easy breath after she came out of the anesthetic. Death occurred on the fourth day, and post-mortem examination showed that she died of acute pulmonary edema, that the kidneys were quite healthy, and that the ureters were quite uninjured and far away as usual from the clamp wire. (I had feared they would not be found to be so.)

This was a lesson which could not be misunderstood, and I immediately published my experience, which received many important confirmations, to the effect that ether had secondary results of an extremely risky kind on the kidneys and on the lungs, but the utterance made but very little impression. My experiences were summarized and published, in 1884, in a paper entitled "A Series of One Thousand Cases of Abdominal Sections," as follows, and I thought then, as I think now, that they were the most important sentences I have ever published in my life :

The question of the best anesthetic for use in abdominal surgery is one to which, of course, I have given a very large amount of attention, and it is very singular that in the class of drugs, the action of which there can be the least doubt about, we are, as yet, certainly very unsettled in our views. Like all pupils of Simpson, I began my professional life with a most profound belief in the advantages of chloroform over all other anesthetics. I have never seen an accident from chloroform, but, partly by reason of the fear of inquests and partly by the example and teaching of Dr. Keith, a belief grew in my mind that ether was preferable to chloroform, and at first I had the impression that the sickness after ether was less marked than after the use of its rivals. I was not, however, very long in discovering that ether has special risks for people with a tendency to bronchitis ; and later on I discovcovered, and have already published the fact, that during the administration of ether the secretion of urine is completely arrested. It was subsequently very forcibly impressed on me that, for patients with damaged kidneys, ether is a dangerous anesthetic, and although I cannot say that I have seen any fatal results arising from this peculiarity of its action, I certainly have had abundant cause to fear it. My first alteration, therefore, in my views concerning ether, was to limit its application to patients under forty, but even after this I found my confidence in its safety greatly diminished by the fatal occurrence of bronchitis in a case of hysterectomy in a woman aged thirty. In this case the patient's breathing was embarrassed from the moment she recovered from the anesthetic, her urine was scanty and became ultimately albuminous, and she died on the fourth day from suffocative catarrh, the post-mortem showing that, so far as the operation was concerned, everything was perfectly satisfactory.

These utterances attracted very little attention, and my example was very little followed, if at all. The reasons were two : First, that crowds of experiments on animals had just been published, in which it was proved that, so far as animals were concerned, ether was a safe anesthetic and chloroform was not. Even if this conclusion were correct, and for many reasons I doubted, and still doubt it, I urged the plea that what was true about animals need not be, and was not, true about human beings, and that no animal was known which suffered from chronic renal degeneration or which died from acute pulmonary catarrh. Nobody would listen to me, and my arguments were put down as those of a crank who had strong views about experimenting on animals. My views on this subject were entirely misrepresented, as they have been again, and quite recently, by the editor of the *British Medical Journal.* I am content to leave the quasi-moral arguments alone, and I confine myself to my own department, that of surgery, and I said fourteen years ago what I say now, that, for surgical purposes, experiments on animals are wholly untrustworthy and have been, in very many instances, grossly misleading. I have never said anything more, and no amount of bullying or ridicule will make me say anything less. So much for the first reason of the neglect with which my utterances on anesthetics were received. My only comfort is that, last year, I induced the Council of the British Medical Association to exclude from their officially conducted research on anesthetics all experiments on animals.

The second reason was a stronger one. England is emphatically the land of coroner's inquests ; and, considering also it is the land which has established the freest and yet most responsible system of jurisprudence the world has yet seen, its people stand an amount of nonsense from coroners and their juries which is most astonishing.

The thing which an English practitioner hates above everything is a coroner's inquest upon any incident in which the conduct of his own practice is concerned may be called in question. Coroners seem always fond of making public inquiry into cases of death under an anesthetic, as there seems to be ingrained in the public mind, from the ridiculous misrepresentations they see on the stage and read of in novels, that anesthetics may be used for purposes of rape and robbery. Every such death is, therefore, blazoned abroad until the use of chloroform has become a *bête noire* of surgical practice alike for practitioner and patients. Chloroform, when it kills, which it does very rarely, kills on the instant, and, in England, there is an inquest. When ether kills, which it does far more frequently, it kills some days after its administration, and there is no inquest, not even an inquiry. In Scotland there are no coroners and no inquests, and as it is universally known and believed there that chloroform is far safer and better than ether, the former is used and the latter is not.

To get over the difficulty, I began to use a mixture, and soon found that it was a great advance over either of the two anesthetics used separately. I vary the proportions according to age, increasing the proportion of chloroform from one-third to two-thirds rapidly after forty, and in case there is any suspicion of renal or pulmonary incompetency.

Twelve years' experience has driven entirely out of my practice all those disasters which ether brought into it. In a number of administrations, now amounting to a great many thousands, not a mistake has occurred, and alarms occur only where some new and inexperienced administrator will indulge in such fantastic tricks as pushing back the tongue by pressing up the jaw, or violating in some other foolish way the simple rules for administration laid down over forty years ago by Simpson, not one of whose methods has yet been surpassed.

I have also this advantage, that when compelled to operate with the assistance of some practitioner in the country, whose opportunities of administering an anesthetic are few and far between, I hand him my anesthetic, with the usual remark that it is an ether mixture; he goes to his work with confidence devoid of fear. If I told him that it was chloroform, his hair would stand on end, he would think of nothing but an inquest all the time, and I should never have the patient properly under from beginning to end of the operation. In fact, I should have all the elements of danger, as Simpson lays them down, arrayed against me.

Further, in discussing details of an operation with women patients, half of them want to know if they must take chloroform, because they are sure their hearts won't stand, and they have been told this, that and the other. "Ether mixture, far better and safer than chloroform," settles the question and calms their morbid imaginations.

Perhaps you will permit me, in another letter, to reply to the third part of Dr. Byron Robinson's question concerning infection. Meantime, accept my assurances of respect and believe me, yours sincerely,

LAWSON TAIT.

THE CRESCENT, BIRMINGHAM, ENG., December 9, 1893.

